# Supplementary material for: Lectin binding and gel secretion within Lorenzinian electroreceptors of Polyodon
Source: PLoS One. 2022 Nov 17;17(11):e0276854. doi: 10.1371/journal.pone.0276854 (PMC9671328; doi:10.1371/journal.pone.0276854)
Supplement: S1 Fig — (DOCX) [file pone.0276854.s001.docx]

“Lectin binding and gel secretion within Lorenzinian electroreceptors of *Polyodon*”

D.F. Russell, W. Zhang, T.C. Warnock, L.L. Neiman


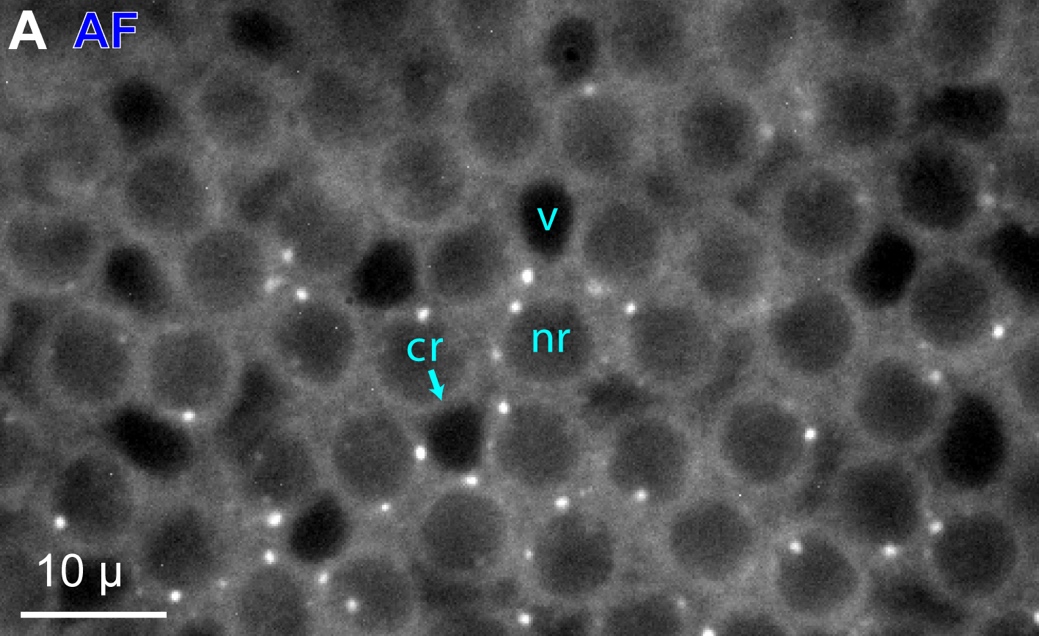


**Supplement S1. Additional image.**

(**A**) Blue autofluorescence (AF) of an unlabeled EN, imaged en face; monochrome camera, 40x 0.75 NA water lens. This single parallel optical section within an EN was contrast-adjusted to show blue autofluorescence from receptor cell cytoplasm (cr, light gray) as thin annular shells surrounding nuclei (nr, dark gray ovals). Dark voids (v) between receptor cells corresponded to non‑autofluorescent support cells. Bright autofluorescent particles of unknown origin had diameters 0.83 ± 0.19 µ (v=36; S2 tab 3).
