# Supplementary material for: Lectin binding and gel secretion within Lorenzinian electroreceptors of Polyodon
Source: PLoS One. 2022 Nov 17;17(11):e0276854. doi: 10.1371/journal.pone.0276854 (PMC9671328; doi:10.1371/journal.pone.0276854)
Supplement: Attachment — Submitted filename: Response_to_reviewer.docx [file pone.0276854.s004.docx]

2022 August 27

Response to reviewer, PONE-D-22-01494 - EMID:a808714d13366f57

Title: “Lectin binding and gel secretion within Lorenzinian electroreceptors of *Polyodon*”

Corresponding author: David F. Russell

1. Preabsorption method: This is now described in detail in the Results text section “Lectin controls”. Also, we moved preabsorption imaging data into Fig 2 instead of ex-supplement 1. A lectin solution was pre-mixed with inhibiting sugar and incubated for 1-2 hours, and then the mixture was applied to a tissue slide so that lectin binding occurred in the presence of inhibiting sugar, as recommended by the reviewer.

The reviewer required stating the concentrations of preabsorption sugars. We now supply this value for N‑acetyl-D-galactosamine (100 mM). We also used a complex sugar mixture, chitin hydrolysate solution (Vector Labs SP‑0090), in other preabsorption controls. However, Vector Labs’ data sheet did not state the concentrations of its several component sugars, including haptenic N‑acetyl-D-glucosamine and various oligomers of it. That is, chitin hydrolysate was a complex degradation mixture from chitin, incompletely characterized but widely used with lectins. We mixed lectins with a low dilution of chitin hydrolysate solution. Therefore, we can only state that the component sugars of chitin hydrolysate were “in excess”.

2. “Chitin is the main component of insects and bacteria covering the outside of body or cells, respectively, but it does

not exist in fish.”

The reviewer keyed on the term ‘chitin’, an arthropod polymer. However, what we used for preabsorption of WGA was chitin hydrolysate solution, consisting of haptenic N-acetyl-D-glucosamine and oligomers of this amino sugar, to which WGA can bind. Chitin was merely the source of the hydrolysate. To clarify this, we substituted “chitin hydrolysate” for “chitin” throughout the text, and also in panel labels of Fig 2.

“WGA actually binds strongly to chitin [N-acetyl-D-glucosamine and oligomers], but WGA ligands in fish cells or tissues thought to be clustered sialic acids attached on O-linked glycans of glycoproteins such as mucins”

The reviewer pointed out that WGA also binds to terminal sialic acid groups, and supplied the original reference for this, now added to the article. We previously cited this issue in Discussion, but now mention it in the Results (page 12) and address it explicitly in paragraphs #1 and #8 of the Discussion. We point out in paragraph #8 that glucosamine is highly concentrated (65% of total carbohydrate) in the gel filling homologous ampullary organs.

“Therefore, the observation that AO gel was stained with WGA or SBA is considered to be a mucin-like secreted proteins.”

We added paragraph #5 to the Discussion citing recent structural analyses [ref 7] on glycoproteins in the gel filling Lorenzinian ampullary organs, which found peptide fragments of mucin-like proteins. However, this need not imply WGA binding to sialic acids on mucin, as the reviewer asserted. Instead, ref 7 indicated sparse sialic acids, but found abundant N-acetyl-D-glucosamine in numerous keratan sulfate sidechains on mucin and other proteins, consistent with N-acetyl-D-glucosamine and its oligomers as a basis for our observed label of *Polyodon*’s ampullary organ gel by WGA.

3. “N‑acetyl-D-galactosamine” and “N‑acetyl-D-glucosamine” are now used uniformly throughout the article.

4. We thank the reviewer for original references, now added to the Discussion, about ligands of lectins RCA and ConA. Our previous Results and Discussion text attempted to compare labeling by these two lectins, but now this has been removed and the related text reorganized, based on our recent lab work.

Following the reviewer’s suggestion, we included part of their commentary as Discussion paragraph #6, in quotation marks.
